# Reactome: a knowledge base of biologic pathways and processes

**DOI:** 10.1186/gb-2007-8-3-r39

**Published:** 2007-03-16

**Authors:** Imre Vastrik, Peter D'Eustachio, Esther Schmidt, Geeta Joshi-Tope, Gopal Gopinath, David Croft, Bernard de Bono, Marc Gillespie, Bijay Jassal, Suzanna Lewis, Lisa Matthews, Guanming Wu, Ewan Birney, Lincoln Stein

**Affiliations:** 1 European Bioinformatics Institute, Wellcome Trust Genome Campus, Hinxton, Cambridge CB10 1SD, UK; 2 Cold Spring Harbor Laboratory, Bungtown Road, Cold Spring Harbor, NY 11724, USA; 3 NYU School of Medicine, First Avenue, New York, NY 10016, USA; 4 College of Pharmacy and Allied Health Professions, St. John's University, Utopia Parkway, Queens, NY 11439, USA; 5 Lawrence Berkeley National Laboratory, Cyclotron Road 64R0121, Berkeley, CA 94720, USA

## Abstract

Reactome http://www.reactome.org, an online curated resource for human pathway data, provides infrastructure for computation across the biologic reaction network. We use Reactome to infer equivalent reactions in multiple nonhuman species, and present data on the reliability of these inferred reactions for the distantly related eukaryote *Saccharomyces cerevisiae*. Finally, we describe the use of Reactome both as a learning resource and as a computational tool to aid in the interpretation of microarrays and similar large-scale datasets.

## Abstract 2

Reactome, an online curated resource for human pathway data, can be used to infer equivalent reactions in non-human species and as a tool to aid in the interpretation of microarrays and other high-throughput data sets.

## Rationale

When the human genome program was first envisioned, it was anticipated that having a catalog of all of the genes in the human body would vastly enhance our knowledge of how these components work together. Although the availability of the genome sequence has indeed given us a powerful tool to improve our understanding of biology, it has revealed the difficulty of deriving the principles of biology from its individual parts. An apt analogy is an attempt to deduce the principles of powered flight, let alone the working details of a modern aircraft, from the components of an Airbus 380 laid out on a hanger floor.

Although the comprehensive genome sequence has only recently been revealed, biologists have been characterizing the roles played by specific proteins in specific processes for nearly a century. Although this information is not comprehensive for any organism, it spans a considerable breadth of knowledge and is sometimes exquisitely detailed. Examples range from the oxidative metabolism of sugar molecules [1], through the molecular control of the cell cycle [2], to the atomic details of selective ion transport [3]. This information is stored as primary literature, review articles, and human memories. It is transmitted between researchers by printed, digital, and oral routes, but it remains largely inaccessible to computational investigation. Much biomedic literature is now available in online form, but attempts to use it for computational analysis must confront the unsolved problems of natural language processing. Hence, if we wish to reason with this information, then we must do so in the traditional way - by collating all information possibly related to the subjects of interest, reading it, and memorizing the relevant parts. In the postgenomic world, however, in which information has been gathered on tens of thousands of genes, proteins, and other potentially relevant biomolecules, the traditional method becomes increasingly difficult to put into practice.

Inability to manipulate this knowledge computationally is most keenly felt in the analysis of high-throughput functional data, where the lack of computationally accessible knowledge interferes with our ability to check the high-throughput data for consistency with what is already known. For example, microarray profiling of insulin-sensitive versus insulin-resistant tissues typically detects expression pattern changes in hundreds of genes [4-6]. Current electronic resources do not allow the list of differentially expressed genes to be automatically cross-checked against well described pathways.

Recent efforts [7,8] have established databases of published kinetic models of biologic processes ranging in complexity from glycolysis to regulation of the cell cycle. These databases allow researchers to browse and visualize pathway models and, in some cases, to run simulations for comparison with experimental data. Of necessity, these databases are limited in scope to a few very well characterized pathways; they are deep but narrow collections. On the other end of the spectrum, interaction databases such as Biomolecular Interaction Network Database (BIND) [9] record the results of high-throughput molecular interaction studies as well as limited literature-based curation of genetic and physical interactions. These databases are broad but shallow; individual interactions have little additional information, and hence are not easily associated with the larger biologic processes in which they participate.

In this paper we present the Reactome knowledge base of biologic processes. Like kinetic model databases, Reactome obtains information from expert bench biologists, and like interaction databases, Reactome strives for comprehensiveness. However, Reactome seeks to provide integrated, qualitative views of entire human biologic processes in a computationally accessible form. Here, we describe the design of Reactome and the operating procedures used to collect, curate, and verify the quality of the contents of the database, and discuss new biologic insights emerging from this process.

## The Reactome data model

At the cellular level, life is a network of molecular interactions. Molecules are synthesized and degraded, transported from one location to another, form complexes with other molecules, and undergo temporary and permanent modifications. However, all of this apparent complexity can be reduced to a simple common representation; each step is an event that transforms input physical entities into output entities.

Much of the power and expressivity of any pathway database lies in the data model used to represent these molecules and their interactions. Reactome uses a frame-based knowledge representation consisting of classes, or 'frames', that describe various concepts such as reaction, pathway, and physical entity. Pieces of biologic knowledge are captured as instances of those classes. Classes have attributes, or 'slots', which hold pieces of information about the instances. For example, each reaction is represented as an instance of the class 'reaction', whose input and output slots are filled with the reactants (input) and products (output) of the given reaction.

The Reactome data model extends the concept of a biochemical reaction to include such things as the association of two proteins to form a complex, or the transport of an ubiquitinated protein into the proteasome. Reactions are chained together by shared physical entities; an output of one reaction may be an input for another reaction and serve as the catalyst for yet another reaction.

It is convenient, if arbitrary, to give such a set of interlinked reactions a name, thereby organizing them into a goal-directed 'pathway'. In Reactome, the reaction in which fructose-6-phosphate is formed from glucose-6-phosphate is followed by a reaction in which fructose-6-phosphate and ATP are transformed into fructose-2,6-bisphosphate and ADP, and another in which - in response to the positive regulatory effect of fructose-2,6-bisphosphate - fructose-6-phosphate and ATP are transformed into fructose-1,6-bisphosphate and ADP. Together, these and subsequent reactions form the 'glycolysis' pathway. Pathways can be part of larger pathways. Reactome represents glycolysis and gluconeogenesis (glucose synthesis) as parts of 'glucose metabolism', which in turn is a part of a larger pathway named 'metabolism of small molecules'. Reactome pathways are cross-referenced to the Gene Ontology (GO) biologic process ontology [10,11].

Reactions that are driven by an enzyme are described as requiring a catalyst activity, modeled in Reactome by linking the macromolecule that provides the activity to the GO molecular function term [10,11] that describes the activity. In addition, the Reactome data model allows reactions to be modulated by positive and negative regulatory factors. When a precise regulatory mechanism ('positive allosteric regulation', 'noncompetitive inhibition') is known, this information is captured.

Reactome reactions act upon 'physical entities'. Entities include proteins, nucleic acids, small molecules, and even subatomic particles such as photons. A physical entity can be a single molecule, such as a polypeptide chain, or an ensemble of components, such as a macromolecular complex.

Part of the challenge of describing biologic processes in computable form is the complexity of the many transformations in molecules that occur during the course of a pathway. Molecules are modified, moved from place to place, or cleaved, or they may take on different three-dimensional conformations. Many of these modifications are critical to the process under consideration; for example, phosphorylation of a protein at a particular amino acid residue may convert it from an inactive form to an active form. The Reactome data model handles these issues by treating each form of a molecule as a separate physical entity. Under this scheme the unphosphorylated and phosphorylated versions of a protein become separate physical entities, and if the protein can be phosphorylated at different residues then each distinct phosphorylation pattern is treated separately. The corresponding phosphorylation process is annotated as a reaction whose input is the unphosphorylated physical entity and whose output is the phosphorylated version.

Because the functions of biologic molecules critically depend on their subcellular locations, chemically identical entities located in different compartments are represented as distinct physical entities. For example, extracellular D-glucose and cytosolic D-glucose are distinct Reactome entities. This allows us to treat transport events as ordinary reactions; glucose transport is a reaction that takes extracellular D-glucose as its input, and produces cytosolic D-glucose as its output. To annotate the subcellular locations of molecules, we use a subset of the GO cellular component ontology [10,11] that has been pruned to remove compartments that overlap with others, such as 'intracellular'.

Reactome also treats molecules that have distinct biologically significant conformational states as separate physical entities. For example, a key event in photoreception in the retina is the photon-triggered isomerization of the rhodopsin 11-*cis *form to the all-*trans *form. In Reactome, each functionally significant rhodopsin isomer can be treated separately.

Physical entities that represent the same chemical in different compartments, configurations, or modifications states share much of the same information, and it would be inefficient and error prone to replicate that information for each entity. It is also desirable to identify all physical entities that share the same basic chemical structure or sequence. Reactome handles this using the concept of a 'reference entity', which captures the invariant features of a molecule such as its name, reference chemical structure, amino acid or nucleotide sequence (when relevant), and accession numbers in reference databases. The data model allows each physical entity to refer to its reference entity, and *vice versa*. For the common case of a protein that has undergone post-translational covalent modification, the Reactome data model records the location and type of the modification using the 'modified residue' class.

Most biologic reactions involve not simple molecules, but large macromolecular complexes, and Reactome treats each complex as a named physical entity. This allows us to describe molecular assembly operations, such as the recruitment of double-strand break repair complex components to the site of DNA damage, as a series of reactions in which the inputs and outputs are intermediates in the formation of the DNA repair complex. In the data model, complexes refer to all of the components that they contain, so that it is possible to fetch all complexes that involve a particular component or to dissect a complex to find the individual molecules that comprise it. In the data model, a physical entity comprised of a single molecule is known as a 'simple entity', whereas entities comprising two or more simple entities belong to the 'complex' class.

Like simple entities, complexes that have catalytic activity are cross-referenced to the GO molecular function ontology. When appropriate, we record which component or molecular domain of the complex has the active site for the activity; this aids in the transfer of knowledge to the GO database, which associates molecular function terms with protein monomers and cannot currently accept information about entire complexes.

There are many cases in which it is convenient to group physical entities together into sets on the basis of common properties. For example, the SLC28A2 plasma membrane nucleoside transporter operates equally well on adenosine, guanosine, inosine, and uridine; these four molecules are interchangeable from the point of view of the transport system [12]. In order to avoid creating four almost identical reactions for these nucleosides, Reactome's data model allows the creation of two 'defined sets' for extracellular and cytosolic nucleosides. SLC28A2-mediated nucleoside transport can then be described as a single reaction that converts the extracellular nucleoside set into the cytosolic set. Defined sets are also used to describe protein paralogs that are functionally interchangeable, equivalent RNA splice variants, and isoenzymes.

Another type of set used by Reactome is the 'candidate set'. This is used when the state of knowledge is incomplete and it is believed that one out of several candidate physical entities is responsible for a particular task. This is used, for instance, to express the assertion that, 'The presence of a particular cyclin-dependent kinase is responsible for this step in cell cycle progression, but we do not know which one.'

Finally, there is an 'open set' class, which is used for cases in which all members of the set cannot be explicitly enumerated. For example, in the RNA transcription pathways, we need to describe reactions that involve all mRNAs but we cannot enumerate all distinct mRNA molecules. Instead, we use an open set named 'mRNA'. As we add distinct mRNA molecules to the database, they become a part of this set, allowing them to be treated simultaneously from the perspective of a generic mRNA subject to transcriptional and splicing reactions, as well as from the point of view of a distinct mRNA that is, for example, under the control of a particular transcriptional factor.

Together, the simple entity, complex, and set classes allow detailed and flexible annotation of physical entities and their interactions. For example, Cdc2 protein (Universal Protein Resource [UniProt]:P06493) can be phosphorylated in the cytosol at threonine-14. The phosphorylated form of Cdc2 is distinct from unmodified Cdc2. Both the phosphorylated and unphosphorylated forms can also be found in complexes with cyclins B_1 _or B_2_. Both of these cyclins are represented by a single distinct entity, and the two of them together are represented collectively by a defined set called 'cyclin B'. The complexes between the cyclins and Cdc2 are represented as two instances of the complex class: one complex consisting of the 'cyclin B' defined set and unphosphorylated Cdc2, and the other consisting of the 'cyclin B' defined set and phosphorylated Cdc2. These complexes then take part in the various reactions of the cell cycle pathway. We can simultaneously create complexes of Cdc2 with individual cyclins if a particular cyclin/Cdc2 complex does something that the others do not.

The use of sets simplifies both the curation and the querying of Reactome. For example, the web query interface allows researchers to search for pathways involving 'cyclin B' and obtain a comprehensive list. Without this functionality, a researcher might have to search serially for each member of the set of entities that together comprise cyclin B.

A critical aspect of the Reactome data model is evidence tracking imposed at every level. Every reaction entered into the knowledge base must be backed up by evidence from the biomedical literature, and documented with appropriate citations. Reactome recognizes two types of evidence: direct and indirect. Direct evidence for a reaction in humans comes from a direct assay on human cells. However, much of current biochemical knowledge has been developed from experiments and observations in nonhuman species. Insights obtained in one species are then projected onto other species on the basis of sequence similarity of genes or proteins between the respective species. When work in one species is used to make inferences about a human pathway, it becomes Reactome indirect evidence.

In practice, we use nonhuman experimental data to document an inferred human biologic process with a two-step process. First, we create a reaction that describes the reaction in the nonhuman species, using physical entities that are appropriate for the organism that was directly assayed, for instance *Drosophila *Notch protein. The papers that describe the experiments used to characterize the nonhuman reaction become the direct evidence for that reaction in the knowledge base. Next, we create an inferred reaction that describes the reaction in human, using human physical entities, for example the four human Notch paralogs. The nonhuman reaction is now used as the evidence to support the inferred human reaction. In this way, the complete chain of evidence is preserved from primary experiment to nonhuman reaction, to the inferred human reaction.

Reactome uses well recognized external identifiers to establish connections with other public biologic databases. In addition to GO terms to describe molecular function, biologic process and subcellular compartment, we use ChEBI (Chemical Entities of Biological Interest [13]) and UniProt [14] to reference small molecules and protein sequences, respectively. These cross-references are mandatory fields in the corresponding Reactome records and are hand checked by Reactome staff. In addition, we automatically cross-reference proteins, genes, reactions, and other objects to a variety of popular external databases, including Entrez Gene [15], Online Mendelian Inheritance in Man (OMIM) [16], and Kyoto Encyclopedia of Genes and Genomes (KEGG) [17] (Table 1). We chose ChEBI and UniProt over other potential reference datasets because these resources are heavily curated to remove redundancy.

**Table 1:** Database cross-references in Reactome

Database	Protein	Gene	Small molecule	Activity	Compartment	Process
UniProt	X^a^					
Entrez Genes		X				
ChEBI			X^a^			
GO				X^a^	X^a^	X^a^
Ensembl		X				
UCSC		X				
KEGG		X	X			
OMIM		X				

^a^Curated cross-references. ChEBI, Chemical Entities of Biological Interest; GO, Gene Ontology; KEGG, Kyoto Encyclopedia of Genes and Genomes; OMIM, Online Mendelian Inheritance in Man; UCSC, UCSC Genome Browser; UniProt, Universal Protein Resource.

The data model includes several classes to describe special cases such as biologic polymers and reactions that occur concurrently within a pathway, as well as utility classes to aid in curation workflow management and the website user interface. There are also classes in the data model that allow us to describe functional submolecular domains in proteins, nucleotide sequences, and other macromolecules.

Pathways and reactions can have attached summations (human-readable text) and illustrations. Summations orient the reader and summarize the process in textbook style. Summations can also be used to add comments that do not fit into the Reactome data model.

## Pathway authoring and curation

**Figure 1:**
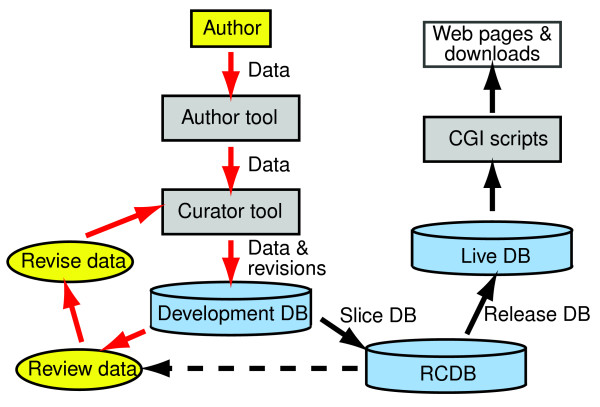
Workflow for authoring and curation of new pathways. Red arrows indicate the part of the process involving interactions between curators and outside experts; black arrows indicate interactions between curators and software engineers. DB, database; RCDB, release candidate database.

All of the information in Reactome comes from expert curation (Figure 1). Reactome curators, who are PhD-level biologists experienced with the data model and authoring tools, together with the Reactome Scientific Advisory Board, identify specific biologic areas to be annotated for Reactome, as well as areas already annotated that warrant revision to incorporate new data. Independent research scientists who are recognized experts in these areas are then recruited to collaborate with Reactome as expert authors. Areas have the scope of journal minireviews, with titles such as 'The TLR3 signaling cascade' and 'Influenza virus packaging and release'. The Reactome data model can accommodate alternative, controversial versions of a single biologic process, as a matter of editorial policy. However, in order to maximize the value of Reactome as a data mining resource for users, experts are asked to construct views of processes that reflect current expert consensus.

**Figure 2:**
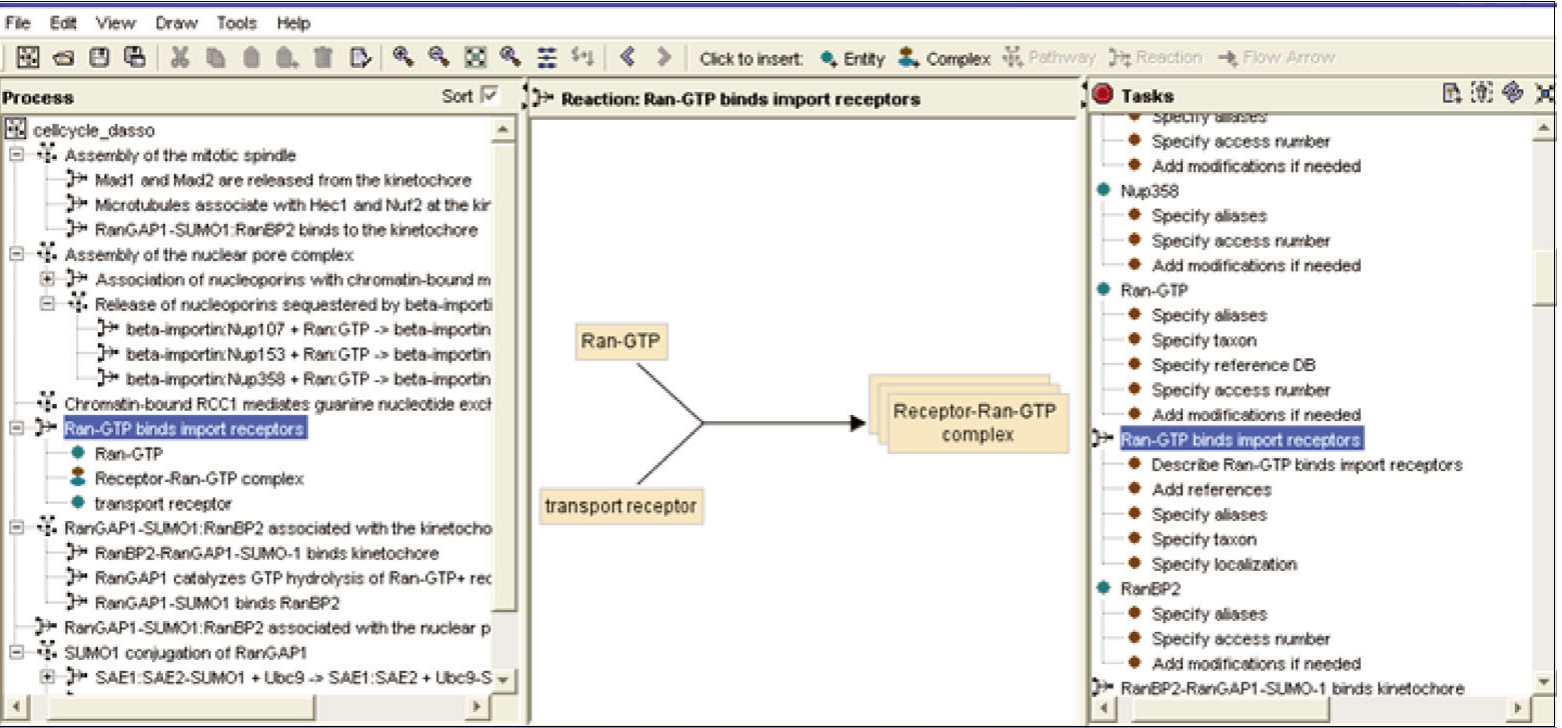
The Reactome author tool provides authors with a graphical user interface to describe pathways and their component reactions in a structured manner.

Typically, the expert and a curator work together to create an electronic outline to define the exact scope of the biologic process to be annotated and to identify and order the reactions that comprise the process. This initial process delimits the biologic area to be annotated, identifying the module that this expert will be authoring. The expert uses a graphical application called the Reactome Author Tool (Figure 2) to add molecular detail to the outline. This detail may include, for example, the identities and subcellular locations of the molecules that participate in each reaction, the role of each molecule (input, output, regulator, or catalyst), the compositions of multimolecular complexes, the order of reactions within a pathway, citations of key primary research publications, and brief free text descriptions of each reaction and pathway. The curator then uses another graphical application called the Reactome curator tool to revise this material and integrate it into the Reactome data scheme. Molecules are linked to their corresponding reference entities and, where appropriate, organized into sets; catalyst activities are linked to GO molecular function terms; and links are created between the new reactions and ones already in Reactome. This information is then uploaded directly from the curator tool into the Reactome development database, so that it can be reviewed by the expert author and other Reactome curators, viewing it on the development version of the Reactome website. The curator then revises the material as appropriate using the curator tool.

Once the content of the module is approved by the author and curation staff, it is peer-reviewed on the development website, by one or more bench biologists selected by the curator in consultation with the author. The peer review is open and the reviewers are acknowledged in the database by name. Any issues raised in the review are resolved, and the new module is scheduled for release.

## The Reactome release process

Reactome follows a quarterly release schedule. The process of creating a release database begins with extracting the finished modules and associated information into a separate 'slice' database (Figure 1). Automated and manual quality assurance procedures are run to check the completeness and consistency of the data. If necessary, material in the development database is revised and a new 'slice' database is generated.

Next, protein orthology mappings are used to computationally predict reactions and pathways in other organisms (this process is described in more detail in the following section). We then add crosslinks to other relevant external resources. After a final round of testing of data and web server testing, the new database is made available via the public website [18].

Reactome has had 19 releases since its first release in 2002. The latest release (November 2006) contains 1,473 curated human proteins, 1,845 reactions, and 691 pathways. This represents roughly 7% of the estimated 21,000 proteins in the human genome [19], and 10% of the roughly 15,000 unique accessions in the human division of UniProt. Reflecting the labor-intensive nature of manual curation, our overall curation rate is roughly 15 new human proteins per curator-month. Our goal, over the next 4 years, is to curate approximately 5,000 proteins manually, as described here, to acquire information about another 5,000 through bulk importation of data from other sources such as protein-protein interaction databases, and thus provide a user at least even odds that a query to Reactome about a human protein will return data.

## Inference of pathways in other species

Since release 4, each Reactome release has included computationally inferred pathways and reactions in multiple nonhuman species, currently *Mus musculus*, *Tetraodon nigroviridis*, *Drosophila melanogaster*, *Caenorhabditis elegans*, *Saccharomyces cerevisiae*, *Aspergillus nidulans*, *Arabidopsis thaliana*, *Dictyostelium discoideum*, *Plasmodium falciparum*, *Escherichia coli*, *Sulfolobus solfataricus*, and 11 others. These species were selected because of the completeness of their genome sequencing and annotation. Together they represent more than 4,000 million years of evolution and span the major branches of life.

The inference process begins with the set of peer-reviewed curated human reactions in the pre-release database. We project these curated reactions onto the genomes of the selected species using protein similarity clusters derived by the OrthoMCL method [20]. Briefly, this method begins with an all-against-all BLASTP performed on all proteins from all the species to be compared. OrthoMCL finds reciprocal best similarity pairs of proteins for each protein and pair of species, as well as 'reciprocal better' similarity pairs within species. The latter are proteins that are more similar to each other within the same species than to any protein in the other species. These pairs are entered into a similarity matrix, normalized by species, and then clustered using a Markov chain length algorithm. The result is sets of related proteins that include both orthologs and recent paralogs that postdate the divergence of the two species.

The next step is the projection of human reactions onto the other selected species. All curated reactions that involve at least one accessioned protein are checked as to whether the proteins involved in that reaction have at least one ortholog or recent paralog (OP) in the other species. Both direct participants in the reaction and enzyme catalysts are considered. In the case of protein complexes, we relax this requirement so that a complex is considered to be present in the other species if at least 75% of its protein components are present in the other species as an OP. Reactions that meet these criteria are considered 'qualified'.

For each qualifying reaction, we create an equivalent reaction for the species under consideration by replacing all protein components with their corresponding OP(s). For proteins with more than one OP in the other species, we create a defined set named 'Homologs of ...' containing the other species' OPs, and use this defined set as the corresponding component of the equivalent reaction.

In the case of complexes that match because of the 75% threshold, some components will have OPs whereas others will not. For those components that do not have an OP, we create placeholder entries in the other species; that is, we infer that a complex exists in the other species that fulfills the same role as the corresponding human complex, but it includes unknown protein components (which might or might not actually exist) as well as those defined by the OP relationships.

Many reactions involve several proteins or complexes. In order to match a putative reaction in another species, all participants - including inputs, outputs, and catalysts - must have a corresponding OP match in the other species.

To create inferred pathways, we connect the newly created reactions in the same way that the original human reactions were. In other words, we infer higher level reactions in the other species as needed in order to replicate the topology of the human pathway. This can cause problems when the presence of a single inferred reaction causes the creation of an entire phantom pathway. For example, cytochrome c (UniProt P99999) is very well conserved across eukaryotes, and so reaction in which this protein is released from mitochondria during apoptotic cell death is inferred in all of these species and causes creation of a pathway, 'apoptosis'. For this reason we are considering implementing a more sophisticated future criterion in which a minimum number of reactions is necessary to create a pathway.

This method of electronically inferring nonhuman reactions via orthology and recent paralogy information has important limitations. Although we assume that a reaction occurs in another species when all proteins involved in the human version of the reaction have an OP in the species, this may not be the case in reality because of diversification of the function of the OP or changes in the tissue or developmental expression pattern. On the other hand we may miss a true corresponding reaction in the other species because the proteins involved may have evolved at the amino acid level while maintaining the same function. Parameters set for the clustering may not fit all biologic ortholog groups.

In order to test the accuracy of our pathway inference procedure, we sought to compare our predictions for an organism evolutionarily distant from humans with the results of expert manual curation in that organism. We took advantage of Yeast Biochemical Pathways (YBP), a set of intermediary metabolism pathways from *S. cerevisiae *independently curated by experts at the Saccharomyces Genome Database (SGD) [21]. These pathways were originally generated using the PathoLogic software, part of the Pathway Tools package [22], from the multispecies pathways database MetaCyc [23].

The focus of YBP is intermediary metabolism, whereas Reactome covers both intermediary metabolism and reactions that involve proteins, large carbohydrates and nucleic acids. To create comparable datasets, we randomly selected 71 curated human reactions from the intermediary metabolism section of Reactome release 17. After removing three equivalent reactions in which the same chemical reaction is catalyzed by paralogous isoenzymes, we were left with 68 reactions in the test set.

We next hand-matched the 68 human reactions to curated reactions in YBP using the YBP web-based query interface. In order to be called a match, the reactants and products of the reactions had to be identical, and the catalysts had to be orthologous to each other by OrthoMCL criteria. Under these criteria, we found that 28 human reactions matched YBP reactions and 31 did not. These 31 reactions included several plasma membrane transport reactions and components of pathways that are highly diverged between fungi and vertebrates. An additional nine YBP entries matched at the reactant level, but the YBP record failed to identify the yeast protein responsible for the reaction's enzymatic activity. This left us with 59 reactions that had a definite YBP match or match absence.

We next ran the standard reaction inference algorithm against the matched and unmatched reactions to yield a total of 27 inferred yeast reactions. Of the 28 hand-matched reactions, the inference algorithm correctly identified 20 reactions and missed four, for a false-negative rate of 28%. The balance of four inferred algorithms correctly inferred the substrates of curated yeast reactions, but they did so by matching the wrong catalyst - often an ortholog whose substrate specificity is known to have changed over the course of evolution. We scored these as false positives. Of the 31 human reactions that did not have an apparent yeast equivalent, the inference algorithms predicted three yeast reactions, which, when combined with the four incorrect catalyst assignments, give a false-positive rate of 22%.

Of the nine human reactions that were hand-matched to incomplete YBP records, the inference algorithm predicted corresponding yeast reactions in five cases, and failed to infer a reaction in four. Because the YBP record was missing information on the catalytic protein, however, we do not know whether these inferences were correct.

From this exercise we estimate that the sensitivity of the inference algorithm is 72% (95% confidence interval ± 15%). The specificity of the inference procedure is 78% (95% confidence interval ± 15%).

We examined the false negatives in more detail. One false negative was the following reaction: 2 glutathione, reduced + H_2_O_2 _⇆ glutathione, oxidized + 2 H_2_O. This reaction is catalyzed by human GPX1, and by the proteins encoded by yeast genes *YKL026C *(GPX1), *YBR244W *(GPX2), and *YIR037W *(GPX3). These three yeast proteins share similarity to human GPX4 and GPX7, but they are not homologous to GPX1. Therefore, this seems to be a case in which the reaction is conserved across the two species, but a different gene encodes the enzyme that catalyzes it.

Another false negative reaction was the following one: hypoxanthine + 5-phospho-α-D-ribose 1-diphosphate ⇆ inosine monophosphate + pyrophosphate. This is catalyzed by hypoxanthine phosphoribosyltransferase (HPRT1) in human and its homolog HPT1 in yeast. These proteins are homologous at the amino acid level, although weakly so, but they are not co-clustered by OrthoMCL. This example would appear to represent a limitation in the OrthoMCL clustering algorithm.

The false positives were also interesting. In four cases, the human reaction does occur in yeast, but it is catalyzed by an enzyme that is different from the one predicted by the inference algorithm. For example, the following reaction occurs in both human and yeast: lysine + α-ketoglutarate + NADPH + H^+ ^→ saccharopine + NADP^+ ^+ H_2_O. YBP indicates that this enzyme is catalyzed by yeast protein LYS1 (*YIR034C*), whereas the Reactome inference predicted the yeast reaction to be catalyzed by LYS9 (*YNR050C*). However, YBP annotates this latter protein as catalyzing the following reaction: glutamate + L-2-aminoadipate 6-semialdehyde + NADPH + H^+ ^→ saccharopine + NADP^+ ^+ H_2_O. This is an apparent case of a change in substrate specificity. Another false positive involved the projection of the following reaction: guanidinoacetate + *S*-adenosylmethionine → creatine + *S*-adenosylhomocysteine. This reaction is not documented by YBP to occur in yeast, but the Reactome inference procedure projected it onto yeast using a homologous enzyme that is annotated as being an arginine methyltransferase that acts on yeast ribosomal protein L12. Finally, in one case, Reactome inferred the following reaction to yeast: 4a-hydroxytetrahydrobiopterin → q-dihydrobiopterin + H_2_O. To do this it used a yeast protein annotated as an open reading frame of unknown function (*YHL018W*). Although it is possible that we have correctly predicted the function of an uncharacterized yeast protein, we consider this unlikely because we were unable to find any literature-based evidence that *S. cerevisiae *metabolizes q-dihydrobiopterin or related molecules.

The list of reactions used in this exercise and their matching YBP entries is available in Additional data file 1.

## Practical applications of Reactome

**Figure 3:**
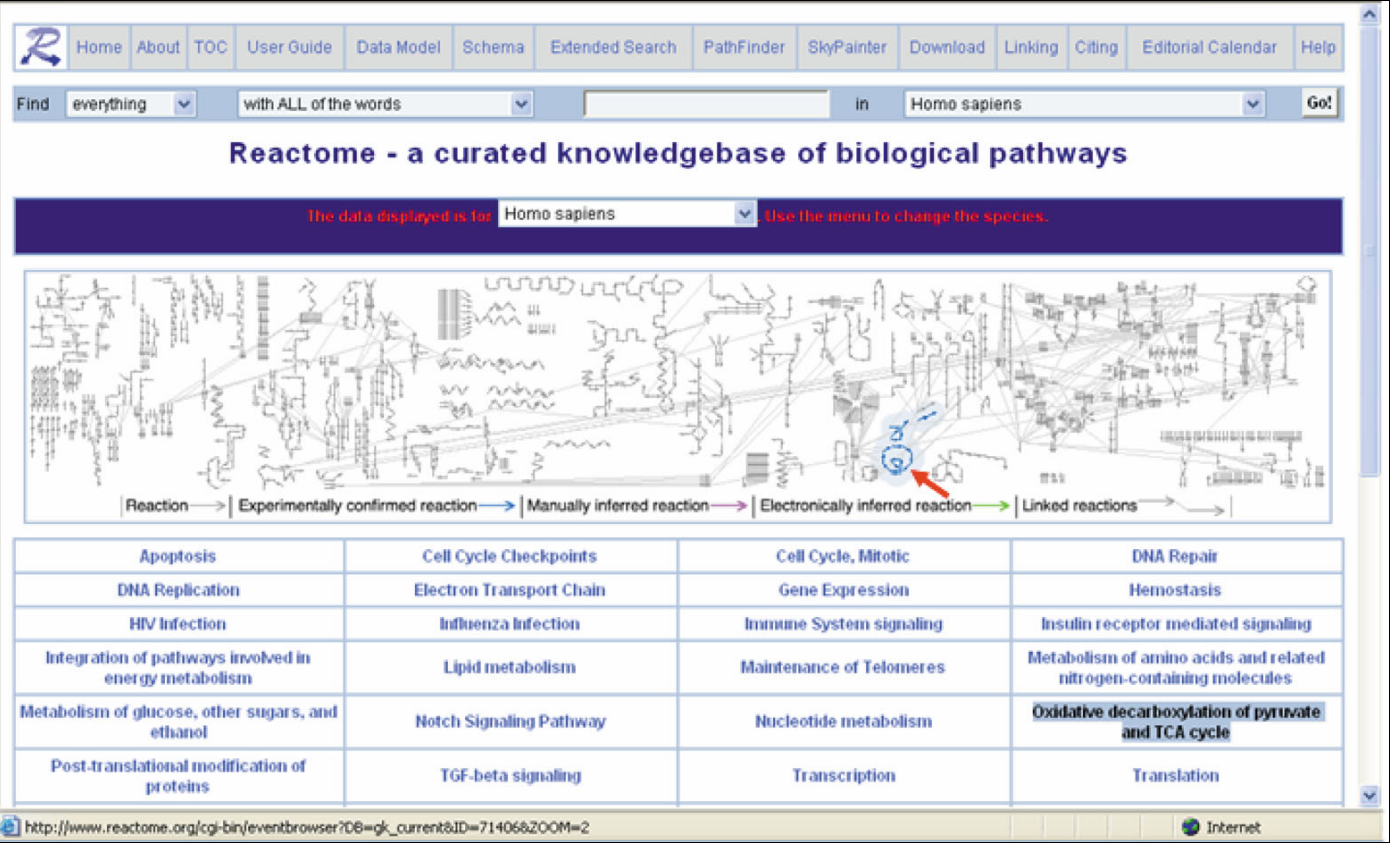
The Reactome home page. The bold arrow in the reaction map at top points to the tricarboxylic acid (TCA) cycle.

The Reactome website [18] can be browsed like an online textbook. The website's front page, shown in Figure 3, features a large 'reaction map' that summarizes all of the currently curated or inferred pathways, and a table of contents that describes each of the top-level pathways in the database. In the reaction map, each reaction is represented as a small arrow, and arrows are joined end to end to indicate that the output of one reaction becomes the input of the next. The reactions are organized in distinctive patterns to allow researchers to become familiar with the different parts of the reaction network. For example, the tricarboxylic acid (TCA) cycle (Figure 3, arrow) is drawn as a circle. As the user moves the mouse over the table of contents, the corresponding reactions in the reaction map are highlighted. Conversely, if the user moves the mouse over the reaction map, the corresponding pathway name is highlighted in the table of contents.

By default, human data are displayed for each top level pathway listed in the table of contents. However, choosing an alternative species from the dropdown menu above the reaction map will take the researcher to the list of pathways that have been inferred in that organism.

**Figure 4:**
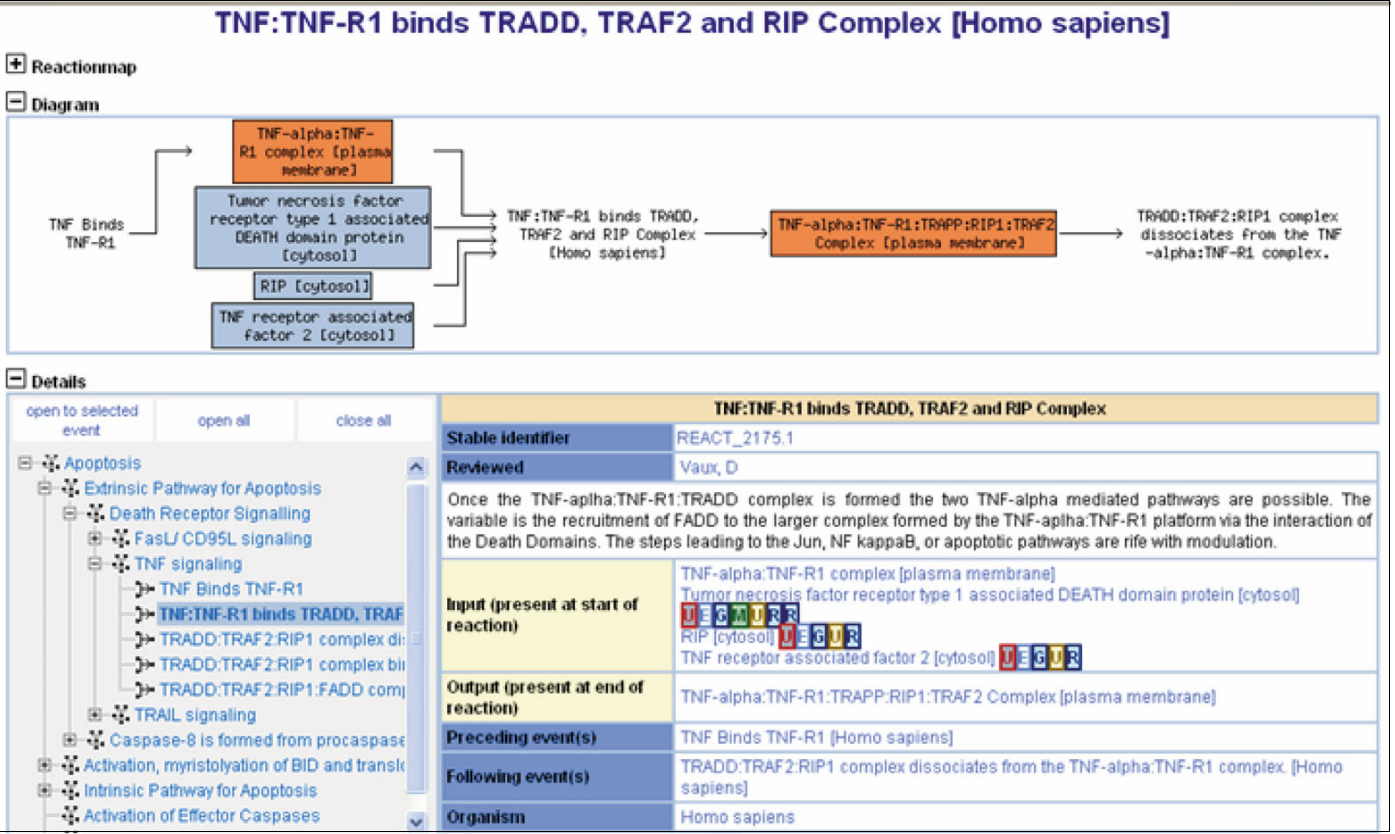
A reaction page.

The researcher can drill down into the database by clicking on a reaction in the reaction map or by clicking on any of the top-level pathways in the table of contents. Pathways are organized in a hierarchy, so that as researchers drill down pathways are described with increasing detail. For example, a researcher who clicks on 'apoptosis' is taken first to a general review of the topic, and shown subtopics for the apoptosis extrinsic pathway, the apoptosis intrinsic pathway, Bid protein activation, and the apoptotic execution phase. Eventually, researchers can drill down to individual reaction pages, such as the one shown in Figure 4, which display the individual components (inputs, outputs, and catalytic activities) of a reaction.

At each level of description, researchers can view the direct or indirect evidence for the pathway or reaction. At the pathway level the evidence is usually a review article that describes the pathway in general terms. At the reaction level, the evidence is one or more citations from the primary literature that confirm the reaction's existence.

From a pathway or reaction page, researchers can download lists of accession numbers for all involved genes and proteins. They can also download a summary of the pathway or reaction in .pdf (human readable), Systems Biology Markup Language (SBML) [24], or Biological Pathways Exchange (BioPAX) level 2 [25] format for computational analysis. SBML is an exchange format commonly used for kinetic modeling of biologic systems, whereas BioPAX is an exchange format designed to describe complex biologic systems.

Pathway and reaction pages are linked to genome databases at UniProt, Entrez Gene, OMIM, and elsewhere (Table 1). A button allows researchers to view the current pathway in the Cytoscape network browser tool [26].

Reactome provides users with the ability to search the database using the name of a reaction, a gene name, a protein name, or any of several other identifiers. For example, to find all reactions involving the human TRAF1 protein, researchers can simply type 'TRAF1' into the search box located at the top of every page. However, more specialized queries are available as well. The most powerful facility is called the 'SkyPainter' - a utility that allows researchers to visualize their own datasets on top of the reaction map. To use the SkyPainter, researchers cut and paste a list of gene identifiers into a web form, or upload a file of identifiers using an upload button. After submitting the form, the SkyPainter uses statistical analysis based on the hypergeometric test [27] to color pathways according to the statistical likelihood that they would contain the listed genes by chance. This highlights those pathways in which the uploaded genes are over-represented.

The SkyPainter recognizes a large number of gene identifiers, including EntrezGene names, accession numbers, and Affymetrix probe sets. It also accepts numeric values, such as expression levels from a microarray experiment. For example, a researcher who is using a microarray to compare a cancerous tissue with a normal control can upload the intensity values from the two experiments to SkyPainter, and it will color the reaction map with red and green to indicate reactions involving genes whose expression is increased or decreased in the malignant cells relative to the normal controls. The SkyPainter can render more complex data, such as a time course series, as an animated movie.

The ortholog-based reaction inference procedure described earlier provides a rough view of how biologic pathways evolve with time. In Reactome release 18, there were 1,784 curated human reactions and 1,450 curated proteins. We projected these onto 12,649 reactions and 17,530 proteins in 22 nonhuman species (Table 2). The probability of successfully inferring a reaction is greatest with closely related species, such as rat, and least with distantly related species, such as *Methanococcus *spp.

**Table 2:** Inferred reactions in target species

Species	Proteins	Complexes	Reactions	Pathways
*H. sapiens*	1450	1,329	1,784	689
*E. histolytica*	570	139	228	193
*D. discoideum*	714	499	598	347
*P. falciparum*	328	235	283	215
*C. merolae*	507	371	470	292
*S. pombe*	619	411	509	321
*S. cerevisiae*	633	401	510	313
*N. crassa*	571	448	601	349
*C. neoformans*	481	334	460	292
*C. elegans*	889	513	693	394
*G. gallus*	1,600	739	1,023	492
*M. musculus*	1,670	1,075	1,376	559
*R. norvegicus*	1,907	981	1,267	547
*T. nigroviridis*	1,358	880	1,135	499
*D. melanogaster*	1,461	646	841	446
*T. pseudonana*	587	377	536	327
*A. thaliana*	1,361	494	596	356
*O. sativa*	1,645	453	562	335
*Synechococcus *spp.	75	60	154	115
*E. coli*	167	131	263	162
*M. tuberculosis*	159	150	273	167
*M. jannaschii*	64	47	112	105
Total nonhuman	17,530	9,473	12,649	6,964

**Figure 5:**
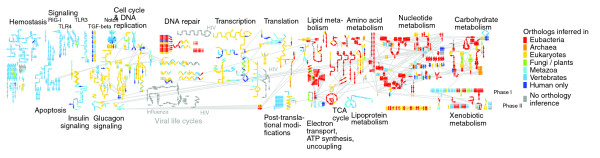
Reactions colored according to the most distant species from *Homo sapiens *in which the reaction could be inferred. Warmer colors indicate reactions found in distantly related species; cooler colors indicate reactions inferred only in closely related species.

The probability of success also varies considerably from pathway to pathway. Figure 5 uses the SkyPainter tool to color the reaction map to represent the most distant species in which we were able to make an inference. Certain pathways, such as polymerase II transcription of mRNA, are highly conserved even among such distant species as the parasitic protozoan *Plasmodium falciparum*. Others, such as the Notch signaling pathway, can only be inferred among metazoans.

One observation that arises from this visualization is that most pathways do not change in a piecemeal manner. Instead, sets of reactions are coordinately gained and lost in a modular way; if one component of a reaction module is absent from a species, then the chances are high that all reactions in the same module will be absent.

Another intriguing observation is a recurrent pattern in which the reactions in the inner core of some pathways are more likely to be conserved across large evolutionary distances, whereas those reactions present at the edges of the same pathways tend to be found only in species closely related to human. This pattern is particularly noticeable in DNA repair, translation, carbohydrate metabolism, and nucleotide metabolism. This observation suggests at least two possible mechanisms. One, as first proposed by Horowitz [28] more than 60 years ago, is that biologic pathways evolve from their centers toward their peripheries. A heterotrophic organism that requires substance A as the input into an essential metabolic pathway will have a selective advantage if it develops the ability to metabolize substances B and C, also present in the environment, into substance A. Hence, as evolution proceeds, core pathways develop novel branches that enable them to use additonal substrates. A second potential explanation for this observation is that the cores of biologic pathways are more constrained than their edges, for example because of increased numbers of regulatory interactions, so that they are exposed to greater degrees of purifying selection than reactions at the periphery. We may be seeing the effects of one or both of these mechanisms.

Because Reactome is heavily curated, we believe it to have a low rate of false reactions. This makes it a good 'gold standard' for training machine learning systems that attempt to infer the presence of genetic or physical interactions from high-throughput datasets. Indeed, Reactome has been used in this way by two independent groups. Ramani and coworkers [29] benchmarked Reactome against several other curated datasets (Human Protein Reference Database, BIND, KEGG, and GO) before selecting training sets for a Bayesian classifier for bimolecular protein interactions. Measured against interactions mined from co-occurrence of gene names in the literature, Reactome had the highest accuracy and was ultimately chosen to train the network. More recently, Franke and coworkers [30] used Reactome as the training set for an application that prioritizes genetic association study gene candidate lists. This system, which also uses a Bayesian framework, identifies common pathways among sets of genes identified by genetic association to a trait of interest.

## Discussion

The concept of a pathway database is, of course, not a novel one. One of the earliest publicly accessible pathway databases dates back to 1992, with the development of EcoCyc [31,32], an online database of the *Escherichia coli *genome and its metabolic pathways. EcoCyc has been followed by a series of metabolic pathway databases based on the EcoCyc infrastructure, including HumanCyc, a database of human pathways [33,34].

Although HumanCyc includes a modest number of curated reactions, much of it is created computationally from sequence similarity on top of an EcoCyc template. Hence, the content of HumanCyc is very much geared toward metabolism. HumanCyc's data model is similar in many respects to Reactome's, and it is built on top of the concept of a reaction that transforms a set of inputs into a set of outputs. The Reactome SkyPainter tool and the HumanCyc Omics Viewer [22] are also similar in design and functionality, aside from the latter's emphasis on metabolic pathways. However, HumanCyc goes beyond Reactome in curating other information about the components of its reactions; for example, it tracks information about the exon structure of genes and their map positions, as well as the chemical structures of small molecules. Reactome, in contrast, links to this information in the appropriate public databases. HumanCyc uses PathoLogic [22] to infer pathways from one species to another. This software implements a Bayesian algorithm that takes pathway topology information into account. Reactome, in contrast, uses a less sophisticated approach that takes the human pathway topology as given and matches individual reactions. As noted above, the Reactome method has the drawback that it can create 'phantom' pathways that contain a single inferred reaction. Unfortunately, in our assessment of Reactome's inference procedures, we were unable to compare our inference algorithm against HumanCyc's because of the fact that PathoLogic inferences were used as the starting material for SGD's pathway curation.

Another popular pathway database is the pathways division of KEGG [17,35], which contains curated metabolic and signaling pathways in species ranging from prokaryotes to humans. KEGG has several important limitations. One is that it uses different data models to represent metabolic and signaling pathways. Although metabolic pathways are represented as chemical reactions, signaling pathways are represented as semantic graphs in which the nodes (molecules or complexes) exert positive or negative influence on other nodes. Signaling pathways thus cannot be connected computationally to metabolic pathways. Another limitation of KEGG is its reliance on Enzyme Commission (EC) numbers to associate metabolic reactions with the physical polypeptides contained in protein and gene databases. This leads to ambiguous, and sometimes incorrect, assignments.

Panther Pathways [36,37] is a curated collection of human pathways with an emphasis on signaling. Panther's data model is based on the Cell Designer application [38,39], which, like Reactome, represents pathways as chemical reactions. Proteins participating in reactions are represented not by single molecules but by sets of proteins, assembled as hidden Markov models. For example, '5HT (5-hydroxytryptamine) transporter' is a set of two human, four mouse, two rat, and two bovine proteins [40]. A second substantive difference between the Reactome and Panther Pathways resources is the curation model; Panther Pathways emphasizes rapid but shallow curation by nonexperts, primarily part-time graduate students and postdoctoral fellows, supplemented by a small number of more senior researchers.

Other human pathway resources include BioCarta project [41], a database of pathway cartoons; GenMAPP [42], a pathway visualization tool; the interaction databases BIND [9], Molecular INTeraction (MINT) [43], and IntAct [44]; the protein databases UniProt [14] and HPRD [45]; descriptive resources such as the Science Signal Transduction Knowledge Environment [46] and the Alliance for Cell Signaling web site [47]; and proprietary products such as the Ingenuity Pathways Knowledge Base [48]. Although these resources often contain extensive data and analysis tools, none of them provides both a publicly accessible internal structure and a data model that allows the full range of human biologic pathways to be represented as computable chemical reactions.

Reactome is distinguished by its uniform treatment of all biologic pathways. It uses the same data model to describe metabolism, signal transduction, DNA replication, the regulation of the cell cycle, and all other biologic processes. This allows Reactome to make connections among these processes. For example, although every pathway database provides the same, correct view of the metabolic steps leading from glucose 6-phosphate to pyruvate, at present only Reactome is able to capture the positive allosteric regulation of the committed step of the pathway, namely conversion of fructose 6-phosphate to fructose 1,6-bisphosphate by fructose 2,6-bisphosphate, and the signaling cascades that link synthesis of the latter compound to levels of the hormones glucagon and insulin. Eliminating artificial distinctions between metabolism, regulatory pathways and higher order reactions makes it possible to write software that computes over the whole biologic reaction network and not arbitrary subdivisions of it.

The known biologic pathways are only a tiny fraction of what goes on in the cell. The next decade will see an ever-expanding flood of biologic information that is likely to overwhelm even the largest curatorial groups. We feel that the way forward is to decentralize and distribute the task of describing pathways in computable and searchable form. To further this vision, we have made Reactome into an open source project. The Reactome data and software are freely available to all users and can be downloaded from the Reactome website [49]. We strongly encourage interested groups to download the Reactome database and software, install it locally, set up their own large or small-scale curatorial effort, and contribute curated pathways back to the main Reactome website for use by the community. In a like manner, we are working with the developers of GenMAPP and other pathway software developers to incorporate support into their applications so that pathways created with these tools can be stored into and retrieved from Reactome databases.

Finally, we encourage other pathway database groups to fully support BioPAX, SBML, and other emerging standards for pathway data exchange, as well as to make use of commonly recognized controlled vocabularies for proteins and small molecules. This will promote the sharing of biologic pathway data among the databases and will speed us toward the ultimate goal of putting all biologic pathway information into a computable form.

## Additional data files

The following additional data are available with the online version of this paper. Additional data file 1 tabulates the results of matching 68 randomly chosen metabolic reactions manually curated in human to the corresponding *S. cerevisiae *reactions inferred using the OrthoMCL-based procedure and corresponding manually curated entries in SGD YBP.

## Supplementary Material

Additional data file 1 The file tabulates the results of matching 68 randomly chosen metabolic reactions manually curated in human to the corresponding *S. cerevisiae *reactions inferred using the OrthoMCL-based procedure (see Results) and corresponding manually curated entries in the SGD YBP. Click here for file
