# Supplementary material for: Reactome: a knowledge base of biologic pathways and processes
Source: Genome Biol. 2007 Mar 16;8(3):R39. doi: 10.1186/gb-2007-8-3-r39 (PMC1868929; doi:10.1186/gb-2007-8-3-r39)
Supplement: Additional data file 1 — The file tabulates the results of matching 68 randomly chosen metabolic reactions manually curated in human to the corresponding S. cerevisiae reactions inferred using the OrthoMCL-based procedure (see Results) and corresponding manually curated entries in the SGD YBP. [file gb-2007-8-3-r39-S1.doc]

**Supplementary Table 1: Reactome inferred yeast reactions compared to curated YBP reactions.**

A total of 68 metabolic reactions manually curated in human were randomly selected from Reactome and used to infer S. cerevisiae reactions using the orthoMCL-based inference procedure described in Results. The same reactions were then hand-matched against corresponding entries in the SGD Yeast Biochemical Pathways (YBP) database and double-checked independently by three observers. The results are categorized into true positives (inferred and matched), false positives (inferred but either not matched or matched with inconsistent catalysts), true negatives (not inferred and not matched), false negatives (not inferred but matched), and uncertain (the YBP entry was missing its catalyst field).

Hyperlinks are as follows:

R-hsa – Reactome web page describing curated human reaction

R-sce – Reactome web page describing inferred yeast reaction

S-rxn – SGD web page describing curated yeast reaction

S-cat – SGD web page describing corresponding catalyst

**1. True positives (matched and inferred, consistent catalyst)**

2'-deoxyadenosine 5'-diphosphate (dADP) + ATP <=> dATP + adenosine 5'-diphosphate (ADP) [R-hsa](http://www.reactome.org/cgi-bin/eventbrowser?DB=test_reactome_17&ID=110645) [R-sce](http://www.reactome.org/cgi-bin/eventbrowser?DB=test_reactome_17&ID=229372) [S-rxn](http://pathway.yeastgenome.org:8555/YEAST/NEW-IMAGE?type=REACTION&object=DADPKIN-RXN) [S-cat](http://db.yeastgenome.org/cgi-bin/locus.pl?sgdid=S000001550)

2'-deoxycytidine + H2O => 2'-deoxyuridine + NH4+ [R-hsa](http://www.reactome.org/cgi-bin/eventbrowser?DB=test_reactome_17&ID=73608) [R-sce](http://www.reactome.org/cgi-bin/eventbrowser?DB=test_reactome_17&ID=229382) [S-rxn](http://pathway.yeastgenome.org:8555/YEAST/NEW-IMAGE?type=REACTION&object=CYTIDEAM-RXN) [S-cat](http://db.yeastgenome.org/cgi-bin/locus.pl?sgdid=S000004235)

2'-deoxyguanosine 5'-diphosphate (dGDP) + ATP <=> dGTP + adenosine 5'-diphosphate (ADP) [R-hsa](http://www.reactome.org/cgi-bin/eventbrowser?DB=test_reactome_17&ID=110676) [R-sce](http://www.reactome.org/cgi-bin/eventbrowser?DB=test_reactome_17&ID=229400) [S-rxn](http://pathway.yeastgenome.org:8555/YEAST/NEW-IMAGE?type=REACTION&object=DGDPKIN-RXN) [S-cat](http://db.yeastgenome.org/cgi-bin/locus.pl?sgdid=S000001550)

2'-deoxyuridine 5'-diphosphate (dUDP) + ADP <=> 2'deoxyuridine 5'-monophosphate (dUMP) + ATP [R-hsa](http://www.reactome.org/cgi-bin/eventbrowser?DB=test_reactome_17&ID=75126) [R-sce](http://www.reactome.org/cgi-bin/eventbrowser?DB=test_reactome_17&ID=229408) [S-rxn](http://pathway.yeastgenome.org:8555/YEAST/NEW-IMAGE?type=REACTION&object=RXN3O-39) [S-cat](http://db.yeastgenome.org/cgi-bin/locus.pl?sgdid=S000003818)

2'-deoxyuridine 5'-diphosphate (dUDP) + ATP <=> deoxyuridine 5'-triphosphate (dUTP) + ADP [R-hsa](http://www.reactome.org/cgi-bin/eventbrowser?DB=test_reactome_17&ID=73637) [R-sce](http://www.reactome.org/cgi-bin/eventbrowser?DB=test_reactome_17&ID=229409) [S-rxn](http://pathway.yeastgenome.org:8555/YEAST/NEW-IMAGE?type=REACTION&object=DUDPKIN-RXN) [S-cat](http://db.yeastgenome.org/cgi-bin/locus.pl?sgdid=S000001550)

3-phospho-D-glycerate <=> 2-Phospho-D-glycerate [R-hsa](http://www.reactome.org/cgi-bin/eventbrowser?DB=test_reactome_17&ID=71654) [R-sce](http://www.reactome.org/cgi-bin/eventbrowser?DB=test_reactome_17&ID=229539) [S-rxn](http://pathway.yeastgenome.org:8555/YEAST/NEW-IMAGE?type=REACTION&object=3PGAREARR-RXN) [S-cat](http://db.yeastgenome.org/cgi-bin/locus.pl?sgdid=S000001635)

5-phosphoribosylamine + glycine + ATP <=> 5-phosphoribosylglycinamide (GAR) + adenosine 5'-diphosphate + orthophosphate [R-hsa](http://www.reactome.org/cgi-bin/eventbrowser?DB=test_reactome_17&ID=73814) [R-sce](http://www.reactome.org/cgi-bin/eventbrowser?DB=test_reactome_17&ID=229599) [S-rxn](http://pathway.yeastgenome.org:8555/YEAST/NEW-IMAGE?type=REACTION-IN-PATHWAY&object=GLYRIBONUCSYN-RXN) [S-cat](http://db.yeastgenome.org/cgi-bin/locus.pl?sgdid=S000003203)

acetoacetyl-CoA+acetyl-CoA => HMG-CoA [R-hsa](http://www.reactome.org/cgi-bin/eventbrowser?DB=test_reactome_17&ID=73918) [R-sce](http://www.reactome.org/cgi-bin/eventbrowser?DB=test_reactome_17&ID=229727) [S-rxn](http://pathway.yeastgenome.org:8555/YEAST/NEW-IMAGE?type=REACTION&object=HYDROXYMETHYLGLUTARYL-COA-SYNTHASE-RXN) [S-cat](http://db.yeastgenome.org/cgi-bin/locus.pl?sgdid=S000004595)

adenosine 5'-diphosphate (ADP) + CTP <=> ATP + cytidine 5'-diphosphate (CDP) [R-hsa](http://www.reactome.org/cgi-bin/eventbrowser?DB=test_reactome_17&ID=110635) [R-sce](http://www.reactome.org/cgi-bin/eventbrowser?DB=test_reactome_17&ID=229933) [S-rxn](http://pathway.yeastgenome.org:8555/YEAST/NEW-IMAGE?type=REACTION&object=CDPKIN-RXN) [S-cat](http://db.yeastgenome.org/cgi-bin/locus.pl?sgdid=S000001550)

adenosine 5'-diphosphate (ADP) + dCTP <=> ATP + 2'-deoxycytidine 5'-diphosphate (dCDP) [R-hsa](http://www.reactome.org/cgi-bin/eventbrowser?DB=test_reactome_17&ID=75124) [R-sce](http://www.reactome.org/cgi-bin/eventbrowser?DB=test_reactome_17&ID=229937) [S-rxn](http://pathway.yeastgenome.org:8555/YEAST/NEW-IMAGE?type=REACTION&object=DCDPKIN-RXN) [S-cat](http://db.yeastgenome.org/cgi-bin/locus.pl?sgdid=S000001550)

alpha-D-glucose 6-phosphate + NADP+ => D-glucono-1,5-lactone 6-phosphate + NADPH + H+ [G6PD tetramer] [R-hsa](http://www.reactome.org/cgi-bin/eventbrowser?DB=test_reactome_17&ID=71293) [R-sce](http://www.reactome.org/cgi-bin/eventbrowser?DB=test_reactome_17&ID=230063) [S-rxn](http://pathway.yeastgenome.org:8555/YEAST/NEW-IMAGE?type=REACTION&object=GLU6PDEHYDROG-RXN) [S-cat](http://db.yeastgenome.org/cgi-bin/locus.pl?sgdid=S000005185)

ATP + alpha-D-glucose => ADP + alpha-D-glucose 6-phosphate [hexokinase 2] [R-hsa](http://www.reactome.org/cgi-bin/eventbrowser?DB=test_reactome_17&ID=70380) [R-sce](http://www.reactome.org/cgi-bin/eventbrowser?DB=test_reactome_17&ID=230667) [S-rxn](http://pathway.yeastgenome.org:8555/YEAST/NEW-IMAGE?type=REACTION&object=R81-RXN) [S-cat](http://db.yeastgenome.org/cgi-bin/locus.pl?sgdid=S000001949) [S-cat](http://db.yeastgenome.org/cgi-bin/locus.pl?sgdid=S000003222)

carbamoyl phosphate + ornithine => citrulline + orthophosphate [R-hsa](http://www.reactome.org/cgi-bin/eventbrowser?DB=test_reactome_17&ID=70560) [R-sce](http://www.reactome.org/cgi-bin/eventbrowser?DB=test_reactome_17&ID=230810) [S-rxn](http://pathway.yeastgenome.org:8555/YEAST/NEW-IMAGE?type=REACTION&object=ORNCARBAMTRANSFER-RXN) [S-cat](http://db.yeastgenome.org/cgi-bin/locus.pl?sgdid=S000003624)

D-ribose 5-phosphate + ATP => 5-phospho-alpha-D-ribose 1-diphosphate (PRPP) + adenosine 5'-monophosphate [R-hsa](http://www.reactome.org/cgi-bin/eventbrowser?DB=test_reactome_17&ID=111214) [R-sce](http://www.reactome.org/cgi-bin/eventbrowser?DB=test_reactome_17&ID=230975) [S-rxn](http://pathway.yeastgenome.org:8555/YEAST/NEW-IMAGE?type=REACTION&object=PRPPSYN-RXN) [S-cat](http://db.yeastgenome.org/cgi-bin/locus.pl?sgdid=S000000164) [S-cat](http://db.yeastgenome.org/cgi-bin/locus.pl?sgdid=S000000901) [S-cat](http://db.yeastgenome.org/cgi-bin/locus.pl?sgdid=S000001003) [S-cat](http://db.yeastgenome.org/cgi-bin/locus.pl?sgdid=S000001664) [S-cat](http://db.yeastgenome.org/cgi-bin/locus.pl?sgdid=S000005422)

GDP + orthophosphate + succinyl-CoA <=> GTP + succinate + CoA [R-hsa](http://www.reactome.org/cgi-bin/eventbrowser?DB=test_reactome_17&ID=71775) [R-sce](http://www.reactome.org/cgi-bin/eventbrowser?DB=test_reactome_17&ID=231248) [S-rxn](http://pathway.yeastgenome.org:8555/YEAST/NEW-IMAGE?type=REACTION&object=SUCCCOASYN-RXN) [S-cat](http://db.yeastgenome.org/cgi-bin/locus.pl?sgdid=S000003476) [S-cat](http://db.yeastgenome.org/cgi-bin/locus.pl?sgdid=S000005668)

L-1-pyrroline-5-carboxylate + NADPH + H+ => proline + NADP+ [R-hsa](http://www.reactome.org/cgi-bin/eventbrowser?DB=test_reactome_17&ID=70664) [R-sce](http://www.reactome.org/cgi-bin/eventbrowser?DB=test_reactome_17&ID=231480) [S-rxn](http://pathway.yeastgenome.org:8555/YEAST/NEW-IMAGE?type=REACTION&object=PYRROLINECARBREDUCT-RXN) [S-cat](http://db.yeastgenome.org/cgi-bin/locus.pl?sgdid=S000000825)

oxaloacetate + glutamate <=> aspartate + alpha-ketoglutarate [mitochondrial] [R-hsa](http://www.reactome.org/cgi-bin/eventbrowser?DB=test_reactome_17&ID=70613) [R-sce](http://www.reactome.org/cgi-bin/eventbrowser?DB=test_reactome_17&ID=231686) [S-rxn](http://pathway.yeastgenome.org:8555/YEAST/NEW-IMAGE?type=REACTION&object=ASPAMINOTRANS-RXN) [S-cat](http://db.yeastgenome.org/cgi-bin/locus.pl?sgdid=S000001589) [S-cat](http://db.yeastgenome.org/cgi-bin/locus.pl?sgdid=S000004017)

pyruvate + glutamate <=> alanine + alpha-ketoglutarate [R-hsa](http://www.reactome.org/cgi-bin/eventbrowser?DB=test_reactome_17&ID=70524) [R-sce](http://www.reactome.org/cgi-bin/eventbrowser?DB=test_reactome_17&ID=231966) [S-rxn](http://pathway.yeastgenome.org:8555/YEAST/NEW-IMAGE?type=REACTION&object=ALANINE-AMINOTRANSFERASE-RXN) [S-cat](http://db.yeastgenome.org/cgi-bin/locus.pl?sgdid=S000004079)

uridine + ATP => uridine 5'-monophosphate + ADP [R-hsa](http://www.reactome.org/cgi-bin/eventbrowser?DB=test_reactome_17&ID=109904) [R-sce](http://www.reactome.org/cgi-bin/eventbrowser?DB=test_reactome_17&ID=232195) [S-rxn](http://pathway.yeastgenome.org:8555/YEAST/NEW-IMAGE?type=REACTION&object=URIDINEKIN-RXN) [S-cat](http://db.yeastgenome.org/cgi-bin/locus.pl?sgdid=S000005295)

UTP + D-glucose 1-phosphate <=> pyrophosphate + UDP-glucose [muscle] [R-hsa](http://www.reactome.org/cgi-bin/eventbrowser?DB=test_reactome_17&ID=71562) [R-sce](http://www.reactome.org/cgi-bin/eventbrowser?DB=test_reactome_17&ID=232219) [S-rxn](http://pathway.yeastgenome.org:8555/YEAST/NEW-IMAGE?type=REACTION&object=GLUC1PURIDYLTRANS-RXN) [S-cat](http://db.yeastgenome.org/cgi-bin/locus.pl?sgdid=S000001518) [S-cat](http://db.yeastgenome.org/cgi-bin/locus.pl?sgdid=S000001004)

**2. False positives (not matched but inferred)**

2'-deoxyadenosine 5'-diphosphate (dADP) + ADP <=> 2'-deoxyadenosine 5'-monophosphate (dAMP) + ATP [R-hsa](http://www.reactome.org/cgi-bin/eventbrowser?DB=test_reactome_17&ID=110135) [R-sce](http://www.reactome.org/cgi-bin/eventbrowser?DB=test_reactome_17&ID=229367)

4a-hydroxytetrahydrobiopterin => q-dihydrobiopterin + H2O [R-hsa](http://www.reactome.org/cgi-bin/eventbrowser?DB=test_reactome_17&ID=71146) [R-sce](http://www.reactome.org/cgi-bin/eventbrowser?DB=test_reactome_17&ID=229559)

guanidinoacetate + S-adenosylmethionine => creatine + S-adenosylhomocysteine [R-hsa](http://www.reactome.org/cgi-bin/eventbrowser?DB=test_reactome_17&ID=71286) [R-sce](http://www.reactome.org/cgi-bin/eventbrowser?DB=test_reactome_17&ID=231333)

**3. False positives (matched and inferred, but inference chooses wrong catalyst)**

5-phosphoribosylglycinamide (GAR) + 10-formyl-tetrahydrofolate => 5'-phosphoribosylformylglycinamide (FGAR) + tetrahydrofolate [R-hsa](http://www.reactome.org/cgi-bin/eventbrowser?DB=test_reactome_17&ID=73813) [R-sce](http://www.reactome.org/cgi-bin/eventbrowser?DB=test_reactome_17&ID=229601) [S-rxn](http://pathway.yeastgenome.org:8555/YEAST/NEW-IMAGE?type=REACTION-IN-PATHWAY&object=GART-RXN) [S-cat](http://db.yeastgenome.org/cgi-bin/locus.pl?sgdid=S000002816)

adenosine 5'-monophosphate (AMP) + ATP <=> adenosine 5'-diphosphate (ADP) + ADP (ADK-1 catalyst) [R-hsa](http://www.reactome.org/cgi-bin/eventbrowser?DB=test_reactome_17&ID=74220) [R-sce](http://www.reactome.org/cgi-bin/eventbrowser?DB=test_reactome_17&ID=230001) [S-rxn](http://pathway.yeastgenome.org:8555/YEAST/NEW-IMAGE?type=REACTION&object=ADENYL-KIN-RXN) [S-cat](http://db.yeastgenome.org/cgi-bin/locus.pl?sgdid=S000002634) [S-cat](http://db.yeastgenome.org/cgi-bin/locus.pl?sgdid=S000000972)

alpha-methyl-acetoacetyl-CoA + CoA <=> propionyl-CoA + acetyl-CoA [R-hsa](http://www.reactome.org/cgi-bin/eventbrowser?DB=test_reactome_17&ID=70844) [R-sce](http://www.reactome.org/cgi-bin/eventbrowser?DB=test_reactome_17&ID=230095) [S-rxn](http://pathway.yeastgenome.org:8555/YEAST/NEW-IMAGE?type=REACTION&object=METHYLACETOACETYLCOATHIOL-RXN) [S-cat](http://db.yeastgenome.org/cgi-bin/locus.pl?sgdid=S000001422)

lysine + alpha-ketoglutarate +NADPH + H+ => saccharopine + NADP+ + H2O [R-hsa](http://www.reactome.org/cgi-bin/eventbrowser?DB=test_reactome_17&ID=70938) [R-sce](http://www.reactome.org/cgi-bin/eventbrowser?DB=test_reactome_17&ID=231530) [S-rxn](http://pathway.yeastgenome.org:8555/YEAST/NEW-IMAGE?type=REACTION&object=1.5.1.7-RXN) [S-cat](http://db.yeastgenome.org/cgi-bin/locus.pl?sgdid=S000001473)

**4. True negatives (neither matched nor inferred)**

2'-deoxycytidine 5'-monophosphate (dCMP) + H2O => 2'-deoxycytidine + orthophosphate [R-hsa](http://www.reactome.org/cgi-bin/eventbrowser?DB=test_reactome_17&ID=109380)

2'-deoxyinosine + ATP => 2'-deoxyinosine 5'-monophosphate + ADP [R-hsa](http://www.reactome.org/cgi-bin/eventbrowser?DB=test_reactome_17&ID=109708)

2'-deoxyuridine 5'-monophosphate (dUMP) + H2O => 2'-deoxyuridine + orthophosphate [R-hsa](http://www.reactome.org/cgi-bin/eventbrowser?DB=test_reactome_17&ID=109481)

2 glutathione, reduced + H2O2 => glutathione, oxidized + 2 H2O [R-hsa](http://www.reactome.org/cgi-bin/eventbrowser?DB=test_reactome_17&ID=71676)

adenosine [extracellular] + 2 Na+ [extracellular] => adenosine [cytosol] + 2 Na+ [cytosol] [R-hsa](http://www.reactome.org/cgi-bin/eventbrowser?DB=test_reactome_17&ID=83952)

adenosine 5'-monophosphate (AMP) + ATP <=> adenosine 5'-diphosphate (ADP) + ADP (ADK-5) [R-hsa](http://www.reactome.org/cgi-bin/eventbrowser?DB=test_reactome_17&ID=110143)

beta-methylglutaconyl-CoA + H2O <=> beta-hydroxy-beta-methylglutaryl-CoA [R-hsa](http://www.reactome.org/cgi-bin/eventbrowser?DB=test_reactome_17&ID=70785)

cytidine [extracellular] + 2 Na+ [extracellular] => cytidine [cytosol] + 2 Na+ [cytosol] [R-hsa](http://www.reactome.org/cgi-bin/eventbrowser?DB=test_reactome_17&ID=83950)

cytidine 5'-monophosphate (CMP) + H2O => cytidine + orthophosphate (5’-nucleotidase, cytosolic III holoenzyme) [R-hsa](http://www.reactome.org/cgi-bin/eventbrowser?DB=test_reactome_17&ID=109448)

cytidine 5'-monophosphate + H2O => cytidine + orthophosphate (5’-nucleotidase cytosolic 1A) [R-hsa](http://www.reactome.org/cgi-bin/eventbrowser?DB=test_reactome_17&ID=73553)

dehydrogenation of testosterone to form 6-dehydrotestosterone [R-hsa](http://www.reactome.org/cgi-bin/eventbrowser?DB=test_reactome_17&ID=76521)

dihydroxyacetone phosphate + D-glyceraldehyde 3-phosphate <=> D-fructose 1,6-bisphosphate [aldolase C] [R-hsa](http://www.reactome.org/cgi-bin/eventbrowser?DB=test_reactome_17&ID=70480)

guanine [cytosol] <=> guanine [extracellular] [R-hsa](http://www.reactome.org/cgi-bin/eventbrowser?DB=test_reactome_17&ID=83998)

guanosine [extracellular] + 2 Na+ [extracellular] => guanosine [cytosol] + 2 Na+ [cytosol] [R-hsa](http://www.reactome.org/cgi-bin/eventbrowser?DB=test_reactome_17&ID=83953)

guanosine 5'-monophosphate (GMP) + H2O => guanosine + orthophosphate (5’-nucleotidase, ecto (CD73) holoenzyme) [R-hsa](http://www.reactome.org/cgi-bin/eventbrowser?DB=test_reactome_17&ID=109285)

guanosine 5'-monophosphate (GMP) + H2O => guanosine + orthophosphate (5’-nucleotidase cytosolic 1A holoenzyme) [R-hsa](http://www.reactome.org/cgi-bin/eventbrowser?DB=test_reactome_17&ID=109398)

hydrolysis of 2'-Deoxythymidine 5'-phosphate to form 2'-deoxythymidine [R-hsa](http://www.reactome.org/cgi-bin/eventbrowser?DB=test_reactome_17&ID=73602)

inosine [extracellular] + 2 Na+ [extracellular] => inosine [cytosol] + 2 Na+ [cytosol] [R-hsa](http://www.reactome.org/cgi-bin/eventbrowser?DB=test_reactome_17&ID=83954)

inosine 5'-monophosphate (IMP) + H2O => inosine + orthophosphate [R-hsa](http://www.reactome.org/cgi-bin/eventbrowser?DB=test_reactome_17&ID=109400)

isomerization of cis,cis-3,6-dodecadienoyl-CoA to form trans,cis-Lauro-2,6-dienoyl-CoA [R-hsa](http://www.reactome.org/cgi-bin/eventbrowser?DB=test_reactome_17&ID=109338)

lauroyl-CoA+FAD<=>2-trans-dodecenoyl-CoA+FADH2 [R-hsa](http://www.reactome.org/cgi-bin/eventbrowser?DB=test_reactome_17&ID=77263)

N-acetylspermine is oxidised spermidine [R-hsa](http://www.reactome.org/cgi-bin/eventbrowser?DB=test_reactome_17&ID=141351)

prostaglandin H2 isomerizes to form Thromboxane A2 [R-hsa](http://www.reactome.org/cgi-bin/eventbrowser?DB=test_reactome_17&ID=76500)

S-(2-methylbutanoyl)-dihydrolipoamide + CoA => alpha-methylbutyryl-CoA + dihydrolipoamide [R-hsa](http://www.reactome.org/cgi-bin/eventbrowser?DB=test_reactome_17&ID=70719)

thymidine 5'-monophosphate (TMP) + H2O => thymidine + orthophosphate [R-hsa](http://www.reactome.org/cgi-bin/eventbrowser?DB=test_reactome_17&ID=109367)

thymine [cytosol] <=> thymine [extracellular] [R-hsa](http://www.reactome.org/cgi-bin/eventbrowser?DB=test_reactome_17&ID=83992)

trans-oct-2-enoyl-CoA+H2O<=>(S)-hydroxyoctanoyl-CoA [R-hsa](http://www.reactome.org/cgi-bin/eventbrowser?DB=test_reactome_17&ID=77333)

uridine [cytosol] <=> uridine [extracellular] [R-hsa](http://www.reactome.org/cgi-bin/eventbrowser?DB=test_reactome_17&ID=83978)

**5. False negatives (matched but not inferred)**

adenylate cyclase produces cAMP [R-hsa](http://www.reactome.org/cgi-bin/eventbrowser?DB=test_reactome_17&ID=111930) [S-rxn](http://pathway.yeastgenome.org:8555/YEAST/NEW-IMAGE?type=REACTION&object=ADENYLATECYC-RXN) [S-cat](http://db.yeastgenome.org/cgi-bin/locus.pl?sgdid=S000003542)

hypoxanthine + PRPP => IMP + PPi [R-hsa](http://www.reactome.org/cgi-bin/eventbrowser?DB=test_reactome_17&ID=73823) [S-rxn](http://pathway.yeastgenome.org:8555/YEAST/NEW-IMAGE?type=REACTION&object=HYPOXANPRIBOSYLTRAN-RXN) [S-cat](http://db.yeastgenome.org/cgi-bin/locus.pl?sgdid=S000002807)

spermine is oxidized to spermidine [R-hsa](http://www.reactome.org/cgi-bin/eventbrowser?DB=test_reactome_17&ID=141341) [S-rxn](http://pathway.yeastgenome.org:8555/YEAST/NEW-IMAGE?type=REACTION&object=SPERMINE-OXIDASE-RXN) [S-cat](http://db.yeastgenome.org/cgi-bin/locus.pl?sgdid=S000004622)

tyrosine + alpha-ketoglutarate => p-hydroxyphenylpyruvate + glutamate [R-hsa](http://www.reactome.org/cgi-bin/eventbrowser?DB=test_reactome_17&ID=71155) [S-rxn](http://pathway.yeastgenome.org:8555/YEAST/NEW-IMAGE?type=REACTION-IN-PATHWAY&object=TYRAMINOTRANS-RXN) [S-cat](http://db.yeastgenome.org/cgi-bin/locus.pl?sgdid=S000001179) [S-cat](http://db.yeastgenome.org/cgi-bin/locus.pl?sgdid=S000003170)

**6. Uncertain (no SGD catalyst given)**

2-acylglycerol + H2O -> glycerol + fatty acid [R-hsa](http://www.reactome.org/cgi-bin/eventbrowser?DB=test_reactome_17&ID=163595) [R-sce](http://www.reactome.org/cgi-bin/eventbrowser?DB=test_reactome_17&ID=229439) [S-rxn](http://pathway.yeastgenome.org:8555/YEAST/NEW-IMAGE?type=REACTION&object=3.1.1.23-RXN)

2'-deoxycytidine 5'-monophosphate (dCMP) + ATP <=> 2'-deoxycytidine 5'-diphosphate (dCDP) + ADP [R-hsa](http://www.reactome.org/cgi-bin/eventbrowser?DB=test_reactome_17&ID=110140) [S-rxn](http://pathway.yeastgenome.org:8555/YEAST/NEW-IMAGE?type=REACTION&object=RXN3O-26)

acetoacetate+succinyl-CoA <=> acetoacetyl-CoA+succinate [R-hsa](http://www.reactome.org/cgi-bin/eventbrowser?DB=test_reactome_17&ID=74177) [S-rxn](http://pathway.yeastgenome.org:8555/YEAST/NEW-IMAGE?type=REACTION&object=3-OXOACID-COA-TRANSFERASE-RXN)

hypoxanthine + D-ribose 1-phosphate <=> inosine + orthophosphate [R-hsa](http://www.reactome.org/cgi-bin/eventbrowser?DB=test_reactome_17&ID=112031) [R-sce](http://www.reactome.org/cgi-bin/eventbrowser?DB=test_reactome_17&ID=231375) [S-rxn](http://pathway.yeastgenome.org:8555/YEAST/NEW-IMAGE?type=REACTION&object=INOPHOSPHOR-RXN)

cytidine + ATP => cytidine 5'-monophosphate (CMP) + ADP [R-hsa](http://www.reactome.org/cgi-bin/eventbrowser?DB=test_reactome_17&ID=73550) [R-sce](http://www.reactome.org/cgi-bin/eventbrowser?DB=test_reactome_17&ID=230878) [S-rxn](http://pathway.yeastgenome.org:8555/YEAST/NEW-IMAGE?type=REACTION&object=CYTIKIN-RXN)

glutamate + NAD+ => alpha-ketoglutarate + NH4+ + NADH + H+ [R-hsa](http://www.reactome.org/cgi-bin/eventbrowser?DB=test_reactome_17&ID=70600) [R-sce](http://www.reactome.org/cgi-bin/eventbrowser?DB=test_reactome_17&ID=231272) [S-rxn](http://pathway.yeastgenome.org:8555/YEAST/NEW-IMAGE?type=REACTION&object=GLUTAMATE-DEHYDROGENASE-(NAD(P)%2B)-RXN)

GTP + Oxaloacetate <=> GDP + phosphoenolpyruvate + CO2 [R-hsa](http://www.reactome.org/cgi-bin/eventbrowser?DB=test_reactome_17&ID=70497) [S-rxn](http://pathway.yeastgenome.org:8555/YEAST/NEW-IMAGE?type=REACTION&object=4.1.1.32-RXN)

thymidine + orthophosphate <=> thymine + 2-deoxy-D-ribose 1-phosphate [R-hsa](http://www.reactome.org/cgi-bin/eventbrowser?DB=test_reactome_17&ID=73613) [S-rxn](http://pathway.yeastgenome.org:8555/YEAST/NEW-IMAGE?type=REACTION&object=THYM-PHOSPH-RXN)

uridine 5'-diphosphate (UDP) + ATP <=> UTP + adenosine 5'-diphosphate (ADP) [R-hsa](http://www.reactome.org/cgi-bin/eventbrowser?DB=test_reactome_17&ID=110726) [R-sce](http://www.reactome.org/cgi-bin/eventbrowser?DB=test_reactome_17&ID=232197) [S-rxn](http://pathway.yeastgenome.org:8555/YEAST/NEW-IMAGE?type=REACTION&object=UDPKIN-RXN)
